# Effects of Nitrogen and Phosphorus on Estuarine Phytoplankton Communities in Aquatic Microcosms

**DOI:** 10.3390/toxics13090798

**Published:** 2025-09-19

**Authors:** Jianan Ling, Chao Wei, Dongning Yang, Jiangning Zeng, Fangping Cheng, Xin Zheng, Zhanhong Yang

**Affiliations:** 1 State Key Laboratory of Environmental Criteria and Risk Assessment, Chinese Research Academy of Environmental Sciences, Beijing 100012, China; ling0jianan@163.com (J.L.); weichao0524@163.com (C.W.); yangdongning24@mails.ucas.ac.cn (D.Y.); 2 Key Laboratory of Marine Ecosystem Dynamics, Second Institute of Oceanography, Ministry of Natural Resources, Hangzhou 310012, China; jiangningz@126.com (J.Z.); fpcheng@sio.org.cn (F.C.); 3 State Key Laboratory of Satellite Ocean Environment Dynamics, Hangzhou 310012, China; 4 Key Laboratory of Nearcoast Engineering Environment and Ecological Security in Zhejiang Province, Hangzhou 310012, China

**Keywords:** aquatic microcosm, phytoplankton, dissolved inorganic nitrogen, reactive phosphate, ecological response thresholds

## Abstract

Phytoplankton serves as the primary producer in estuarine ecosystems, with its community structure and dynamics being directly influenced by the concentration and ratio of nitrogen (N) and phosphorus (P) nutrients. This study utilized raw water from the Yangtze Estuary to establish a series of ocean microcosm systems, setting up gradients of dissolved inorganic nitrogen (DIN) and reactive phosphate (SRP) concentrations to explore the reaction of phytoplankton communities over 30 days. The results indicated that total phytoplankton abundance significantly increased under prolonged exposure to high concentrations of DIN and SRP. However, the community diversity indices exhibited a declining tendency, indicating a simplification and increased instability of the community structure. Diatoms and dinoflagellates, the predominant phytoplankton taxa, differed in their response to DIN and SRP. Diatom abundance rose at elevated DIN concentrations and initially increased and then decreased at high SRP concentrations, while dinoflagellate abundance diminished at high DIN concentrations and persisted in increasing at elevated SRP concentrations. An ecological threshold is the critical point at which the structure or function of an ecosystem undergoes significant changes when subjected to external disturbances or internal changes. The Threshold Indicator Taxa Analysis (TITAN) was employed to identify indicator species within the microcosm systems, revealing that the ecological response thresholds of phytoplankton communities to DIN and SRP were 0.50 mg/L and 0.030 mg/L, respectively. This study quantitatively analyzed the environmental exposure concentrations of DIN and SRP at the community level and calculated the ecological response thresholds, providing fundamental data and a scientific basis for nitrogen and phosphorus management in estuaries.

## 1. Introduction

Rapid coastal urbanization, along with industrial and agricultural activities, has led to substantial nitrogen (N), phosphorus (P), and other nutrient influx into estuarine regions via rivers. This has resulted in significant nutrient overabundance in estuarine waters, which adversely affects the structure and function of the estuarine ecosystem, posing a grave threat to ecological security, marine aquaculture, and human health [1,2]. Phytoplankton serves as the primary producer in estuarine ecosystems, and its community structure and dynamic alterations directly influence the material cycle and energy flow of the entire ecosystem [3,4]. The composition and succession of phytoplankton communities are influenced by the interplay of light, temperature, salinity, nutritional salts, and zooplankton predation [5,6,7]. Nutrient salts, including nitrogen and phosphorus, are essential elements for phytoplankton growth; their concentration, proportion, and availability influence the growth rate, species composition, and community structure of phytoplankton [8,9]. In estuaries, excessive nutrient salt influxes foster algal blooms, which subsequently instigate harmful algal bloom events. These events deplete dissolved oxygen, leading to hypoxia in the benthic zone, disrupt the ecological equilibrium, jeopardize the survival of aquatic organisms, and potentially impact human health via the food chain [10,11,12]. Considering the sensitivity of phytoplankton communities to variations in nutrient salts and their pivotal role in aquatic ecosystems, it is essential to investigate the environmental impacts of nitrogen and phosphorus nutrient salts at the community level. This facilitates a comprehensive understanding of the mechanisms behind phytoplankton community evolution influenced by nutrient salts, while also offering a scientific foundation for the conservation and management of estuarine environments. Numerous studies have investigated the intricate relationship between phytoplankton communities and nitrogen and phosphorus nutrient salts; however, the majority depend on data derived from on-site monitoring and surveys, which are invariably influenced by seasonal variations, weather conditions, and other environmental factors, potentially compromising the accuracy and interpretive validity of the research findings. By establishing a series of microcosm systems, the concentration of nutrient salts, species, and their ratios can be meticulously regulated, allowing for the observation and documentation of the phytoplankton community’s response process, thereby elucidating the inherent relationship between nutrient salts and phytoplankton community structure.

A microcosm is an artificially constructed miniature ecosystem that integrates significant elements of natural ecosystems and biological processes. It examines the ecological impacts of foreign chemicals, commonly termed pollutants, on biological populations, communities, and ecosystems, and is recognized as a model ecosystem [13]. Nutrients are typically not classified as pollutants. Nonetheless, they may be classified as pollutants when their concentration surpasses the environmental carrying capacity, resulting in ecosystem imbalance, degradation of water quality, jeopardizing aquatic life, and potentially threatening human health [14]. Huang Wei et al. examined the phytoplankton community’s response to nitrogen–phosphorus ratios in the surface waters of the East China Sea utilizing microcosm technology [15]. They discovered that variations in nitrogen–phosphorus ratios significantly influenced species richness, Chlorophyll a (Chl a) content, and the ratio of diatoms to dinoflagellates, among other factors [15]. Liu et al. investigated the impact of nutritional salts on the growth of algae and bacteria, revealing that under conditions of nitrogen surplus, algal growth was constrained by phosphorus availability. Bacteria exhibited more competitiveness for phosphorus resources than algae [16]. Tsiola et al. conducted an in-depth study on the impact of nutrient constraints on eukaryotic and prokaryotic species in the Cretan Sea. The findings indicated that the majority of eukaryotic phytoplankton were simultaneously constrained by nitrogen and phosphorus, whereas several heterotrophic prokaryotes were restricted by distinct nutritional salts [17]. Several investigations on the impact of nutrients on aquatic ecosystems have employed microcosm methodologies; however, less research has concentrated on the quantitative correlation between nitrogen and phosphorus levels and phytoplankton within the microcosm. Baker and King introduced the Threshold Species Indicator Method (TITAN) to detect the change points of individual taxa in frequency and abundance and investigate the multiple taxa’s synchronous responses to a slight change in nutrient enrichment gradient [18,19]. The TITAN approach is widely utilized to identify thresholds and indicator species in natural aquatic ecosystems and can assess the community response of phytoplankton to nitrogen and phosphorus in aquatic microcosm systems [20,21,22]. By quantifying the response of species to environmental changes, the TITAN method not only reveals the extent to which species are affected by environmental factors but also elucidates their sensitivity and adaptation to these changes.

This study concentrated on two primary nutrients, dissolved inorganic nitrogen (DIN) and reactive phosphate (SRP), and developed a series of indoor seawater microcosm systems to quantify the ecological effects of nitrogen and phosphorus on phytoplankton at environmentally relevant concentrations and to determine ecological response thresholds. This will aid in comprehending the function of nutrients in estuarine ecosystems and assist in devising targeted pollution avoidance strategies to foster the healthy recovery and sustainable growth of these ecosystems.

## 2. Materials and Methods

### 2.1. Construction of Aquatic Microcosm Systems

**Figure 1:**
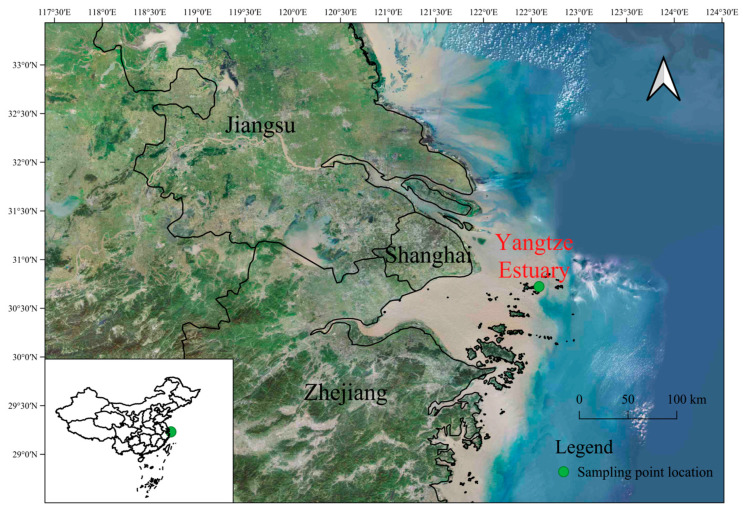
In situ seawater sampling map of the Yangtze Estuary area.

The Yangtze Estuary, located in the eastern part of China, is the third-largest estuary in the world. With the rise of urban agglomeration in the Yangtze Delta, the nitrogen and phosphorus fluxes transported by the Yangtze River continue to increase due to industrial pollution, agricultural pollution, and human activities, leading to increasing eutrophication and deteriorating ecological environment in the Yangtze River Estuary and adjacent waters, becoming the most severely polluted coastal waters in China [23]. This situation in the Yangtze Estuary is not unique; it is a typical example of what is happening in many estuaries around the world where urbanization and industrialization are advancing rapidly. Natural surface seawater (containing plankton communities) was collected from the Yangtze River Estuary (30°43′25″ N, 122°34′58″ E) (Figure 1), and its physicochemical parameters were determined in accordance with the Specification for Marine Monitoring (GB 17378.4-1998) [24]. The original water-quality state was as follows: DIN concentration is 0.398 mg/L, SRP concentration is 0.010 mg/L, DO (dissolved oxygen) concentration is 6.87 mg/L, pH value is 8.15, and Chl a concentration is 0.02 μg/L. Prior to the experiment, large zooplankton were eliminated by filtering through a 200 μm mesh, and the aquatic microcosm systems were established in polyethene aquariums (Zhoushan Base of the Second Institute of Oceanography, Ministry of Natural Resources, diameter: 50 cm, height: 50 cm, volume: 100 L), with each tank receiving 45 L of seawater.

Throughout the experiment, the indoor temperature was maintained at 22.0 ± 1.0 °C, natural light was simulated using fluorescent lamps, with a photoperiod of 12 h of illumination and 12 h of darkness. This work established various gradients of DIN and SRP concentrations to replicate differing nutrient levels, utilizing the maximum nitrogen and phosphorus values recorded in the Yangtze Estuary and the Chinese seawater quality requirements (GB 3097-1997) [25] (refer to Table 1 and Table 2). The experiment sustained the predetermined N and P concentrations by incorporating sodium nitrate-nitrogen (NaNO_3_-N) and sodium dihydrogen phosphate-phosphorus (NaH_2_PO_4_-P) stock solutions. The experiment spanned 30 days, commencing with a 3-day stabilization period, followed by measurements of DIN, SRP, Chl a, and phytoplankton biomass every 3 days. According to the measurement results of DIN and SRP, supplement the required nutrients in the box to ensure that the concentration of nutrients in the water remains stable at the present level during the experiment.

### 2.2. Monitoring of Water Quality Parameters

Sampling and Analysis of DIN and SRP: A total of 250 mL of water sample was collected from various locations inside each tank and subsequently filtered using a 0.45 μm cellulose acetate membrane. The concentrations of DIN, comprising NO_3_^−^-N, NO_2_^−^-N, and NH_4_^+^-N, as well as SRP, were quantified in accordance with the Marine Survey Specifications (GB 17378.4-1998) [24]. The precise analytical methods were as follows: NO_3_^−^-N was determined using the zinc–cadmium reduction method, NO_2_^−^-N was determined through the diazotization-azo coupling method, NH_4_^+^-N was determined utilizing the sodium hypobromite oxidation method, and PO_4_^3−^-P was determined employing the phosphomolybdate blue method.

Sampling and Quantification of Chl a: We collected 500 mL water samples from multiple areas of each aquarium and incorporated magnesium carbonate suspension to avert acidification and pigment disintegration. Each sample was filtered through a 0.45-micron glass-fiber membrane. The pigment was extracted with 90% acetone at 4 °C in darkness for 24 h, and the extract was centrifuged at 3000–4000 r/min in a high-speed refrigerated centrifuge (HT180R, Shanghai Metash Instruments Co., Ltd., Shanghai, China). Finally, we quantified the extract with a spectrophotometer (UV757CRT, Shanghai Chuoding Analytical Instrument Co., Ltd., Shanghai, China).

The mass concentration of Chlorophyll a in the test solution (mg/L) is calculated according to the equation below [26]:

(1) $$\rho_{i}=11.85\times \left(A_{664}-A_{750}\right)-1.54\times \left(A_{647}-A_{750}\right)-0.08\times \left(A_{630}-A_{750}\right)$$

where $\rho_{i}$—mass concentration of Chlorophyll a in the test solution, mg L^−1^; $A_{664}$—absorbance of the test solution at 664 nm; $A_{647}$—absorbance of the test solution at 647 nm; $A_{630}$—absorbance of the test solution at 630 nm; $A_{750}$—absorbance of the test solution at 750 nm.

The mass concentration of Chlorophyll a in the sample (μg/L) is calculated according to the equation below:

(2) $$\rho =\left(\rho_{i}\times V_{i}\right)/V$$

where ρ—mass concentration of Chlorophyll a in the sample, μg/L; $\rho_{i}$—mass concentration of Chlorophyll a in the test solution, mg L^−1^; $V_{i}$—final volume of the test solution, mL; V—volume of the sample taken, L.

### 2.3. Phytoplankton Sampling and Identification Methods

Biological samples were combined in the tank and extracted utilizing a 250 mL syringe attached to a rubber tubing. Every three days, a 250 mL water sample was extracted from the aquarium and preserved with a 1.5% Lugol’s iodine solution. After leaving the sample to rest for 48 h, the supernatant was removed, and the sample was reprepared using 1% formalin. Subsequently, the samples were identified at the lowest taxonomic level (species or genus) utilizing an inverted microscope (BX19-HK830, Shenzhen Aosvi Optical Instrument Co., Ltd., Shenzhen, China) and classified at the phylum level.

### 2.4. Statistical Methods and Data Analysis

#### 2.4.1. Assessment of Phytoplankton Community Abundance and Diversity

(1) Phytoplankton abundance

The formulae for phytoplankton abundance are shown below [27]:

(3) $$N\;=\;\frac{C_{s}}{F_{s}\times F_{n}}\times \frac{V}{U}\times P_{n}$$

where $C_{S}$—the area of the counting frame (mm^2^); $F_{S}$—the area of each field of view (mm^2^); $F_{n}$—the number of counting fields of view; V—the volume of concentrated samples (mL); U—the counting frame volume; $P_{n}$—the total number of phytoplankton individuals.

(2) Diversity of the phytoplankton community

The formulae for the Shannon-Wiener Diversity Index (H), Pielou’s Evenness Index (J), and Margalef’s Richness Index (M) are shown below:

(4) $$H=P_{i}log_{2}P_{i}$$

(5) $$J=\frac{H}{ln_{s}}$$

(6) $$M=\frac{S-1}{log_{2}N}$$

where $P_{i}$—the ratio of the number of individuals of the species to the total number of individuals, calculated by the formula $P_{i\;}=\;n_{i}/N$, where $n_{i}$—the number of individuals of the species, *N*—the total number of individuals, and *S*—the number of species.

#### 2.4.2. Statistical Methods

(1) Principal Response Curve analysis (PRC)

Assessment of the overall impact of nutrient concentration changes on phytoplankton community composition was performed utilizing the principal response curve (PRC) method. The PRC, a multivariate statistical analytic technique, is frequently employed to evaluate the long-term impacts of contaminants on biological populations at the community level. Its specific appropriateness for analyzing microcosm experimental data is attributed to its capacity to efficiently evaluate community-level data collected at various time intervals [28]. Through redundancy analysis and the inclusion of time as a covariate, PRC enhances the comprehension of community alterations [29]. This method is precise and intuitive, able to visually represent the effects of pollutants on communities over time, which is crucial for assessing long-term alterations in community structure. The regression coefficient Cdt in PRC indicates the variability in alterations of community organization. The treatment effects and alterations in community species composition are illustrated via multivariate time–response graphs. The control group exhibits a linear growth over time, while variations from this trend in the treated groups signify the treatment effects. In the PRC, species weights indicate the extent of association with the principal response curve, while species scores (bk) near 0 imply minimal or no response to the PRC. Significance was evaluated via 999 iterations of Monte Carlo permutation tests. Data were transformed using ln(ax + 1) to satisfy the assumptions of normalcy. PRC analysis and visualization were conducted utilizing the R programming language (R version 4.2.2).

(2) Threshold Species Indicator Method (TITAN)

The TITAN method classifies samples into two categories according to predictor variables to optimize the negative or positive correlation of each taxon with the partition. Indicator values (IndVals) quantify correlation, derived from the abundance change points of all species along the nutrient concentration gradient, and evaluated for uncertainty by permutation tests. IndVal scores were normalized to z-scores, and the totals for affirmative and negative replies were computed. The apex of the cumulative z-scores signifies the community threshold. The reliability of the indicator, purity, and uncertainty of individual taxa and community transition points were evaluated by bootstrap testing (1000 iterations) [18]. Before data processing, species abundances were converted using log_10_(x + 1), and those species occurring more than three times were preserved. This study further establishes ecological response thresholds based on the positive and negative response thresholds obtained using the TITAN technique. The determination of positive and negative response thresholds is fundamentally reliant on the development and reproduction of tolerant and sensitive species at varying nutrient concentrations [30]. When nutrient concentration surpasses the negative response threshold, sensitive species will experience considerable stress, leading to a steady decline in their abundance. As nutrient concentration increases, species with greater nutritional tolerance gradually dominate and establish a predominant role in the community structure. Nonetheless, once the nutrient concentration attains the positive response threshold, even the most resilient species will be significantly impacted, severely restricting their growth and reproductive potential. Conversely, when nutrient content falls below the negative response threshold, the structure of the phytoplankton community may remain relatively stable. Given that sensitive species are integral to community structure, their response to nutrients will affect the entire phytoplankton population, instigating a sequence of structural and functional alterations. Consequently, to safeguard the phytoplankton population from nutritional disturbances, the negative response threshold is frequently employed as the ecological response threshold in the environment. TITAN processing and visualizations were executed utilizing the “TITAN2” package in the R programming language (R version 4.2.2).

## 3. Results

### 3.1. Community Effects of DIN on Phytoplankton

**Figure 2:**
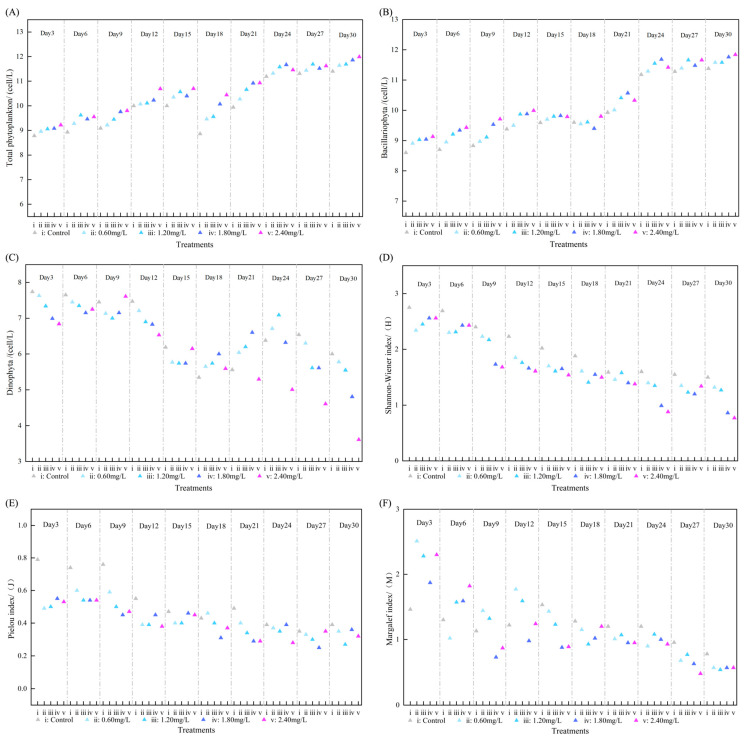
The influence of DIN on the total abundance of phytoplankton (**A**), diatom abundance (**B**), dinoflagellate abundance (**C**), Shannon–Wiener Diversity Index (**D**), Pielou’s Evenness Index (**E**), and Margalef’s Richness Index (**F**) is depicted in figure DIN.

**Figure 3:**
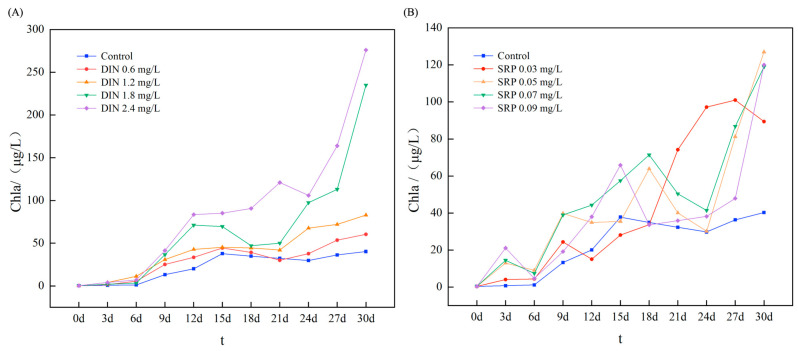
The impact of SRP (**A**) and SRP (**B**) on the Chlorophyll a (Chl a) content of phytoplankton.

During the cultivation period, 88 species of phytoplankton were identified from all collected samples, encompassing diatoms, dinoflagellates, cyanobacteria, and green algae. Diatoms and dinoflagellates comprised 99% of the abundance and represented the predominant phylum of the marine phytoplankton community, aligning with other surveys [31,32]. As shown in Figure 2 and Figure 3, the experiment revealed an increase in the overall abundance of phytoplankton in the treatment group with rising DIN concentration; however, growth decelerated after 15 days and stabilized after 27 days. This suggests that even under extreme DIN circumstances, the proliferation of phytoplankton did not increase significantly. The impact of DIN on diatoms and dinoflagellates varied. The proliferation of diatoms was markedly enhanced with rising DIN concentrations, but the population of dinoflagellates diminished over time, particularly at elevated DIN levels. This indicates that varying concentrations of DIN significantly influence the composition and dynamics of the phytoplankton population. The concentration of Chl a in the treatment group exhibited a substantial increase relative to the control group. As the DIN concentration escalated from 0.6 mg/L to 2.4 mg/L, the Chl a content surged by 49.88% to 584.86% relative to the initial level, with a more pronounced rise observed in the high-dosage group. During the initial phase of the experiment (beginning on the 3rd day), phytoplankton exhibited rapid growth attributable to ample nutrients, and the concentration of Chl a demonstrated a linear growth trajectory. Nonetheless, development decelerated after the 12th day, perhaps because of heightened interspecies competition for dissolved inorganic nitrogen (DIN) and habitat. Following the 21st day, the growth trend was reinitiated, perhaps due to the stabilization of community structure and the attainment of a new equilibrium in interspecies competition.

### 3.2. Community Effects of SRP on Phytoplankton

**Figure 4:**
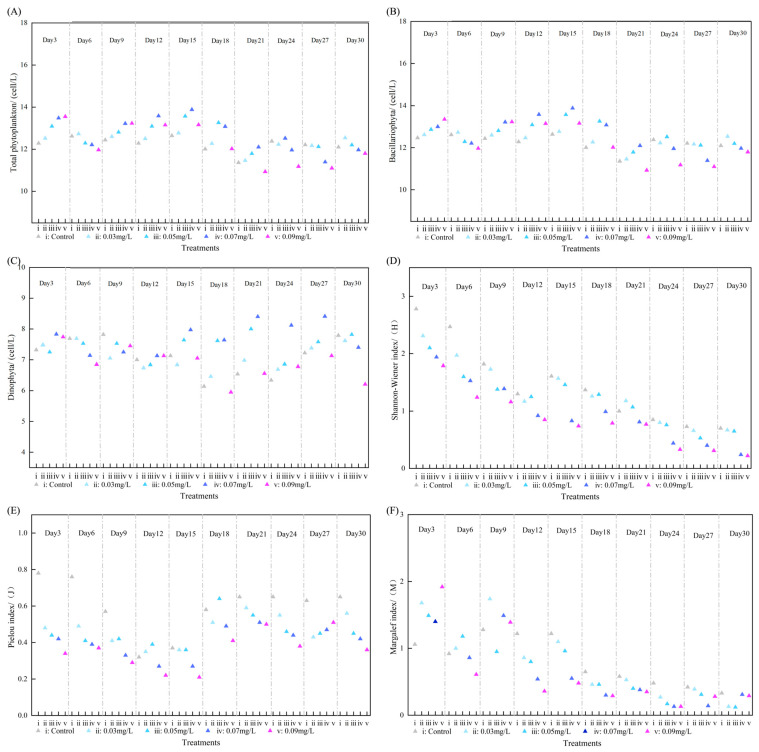
The influence of SRP on the total abundance of phytoplankton (**A**), diatom abundance (**B**), dinoflagellate abundance (**C**), Shannon–Wiener Diversity Index (**D**), Pielou’s Evenness Index (**E**), and Margalef’s Richness Index (**F**) is depicted in figure SRP.

A total of 83 phytoplankton species were identified from all samples taken during the incubation period. Diatoms and dinoflagellates comprised 99% of the total abundance. Figure 4 illustrates that the total abundance of phytoplankton first rose with increasing SRP concentration but then decreased after 24 days, suggesting that SRP initially facilitated phytoplankton growth but later inhibited it under prolonged or high-concentration exposure. The proliferation of diatoms escalated with increasing SRP concentration initially but subsequently diminished at elevated concentrations after 24 days, indicating that high SRP levels may impede diatom growth. The alteration in dinoflagellate abundance diverged from the overall abundance; it rose after 12 days with elevated SRP concentration, particularly at high levels, suggesting that elevated SRP concentrations facilitate dinoflagellate proliferation and may enhance their community proportion. The concentration of Chl a in the treated group was markedly elevated compared to the control group, suggesting that SRP facilitated phytoplankton proliferation. When the SRP concentration ranged from 0.03 to 0.09 mg/L, the Chl a content increased by 121.84% to 215.14%. This indicates that even minimal concentrations of SRP can substantially influence phytoplankton development, consequently altering community structure. The experiment demonstrated that the elevation of SRP resulted in heightened competition among phytoplankton and community succession, with these alterations exhibiting considerable temporal variations, thereby illustrating the dynamic responses of various populations to SRP and their interrelationships.

### 3.3. Effects of DIN on Phytoplankton Community Structure

**Figure 5:**
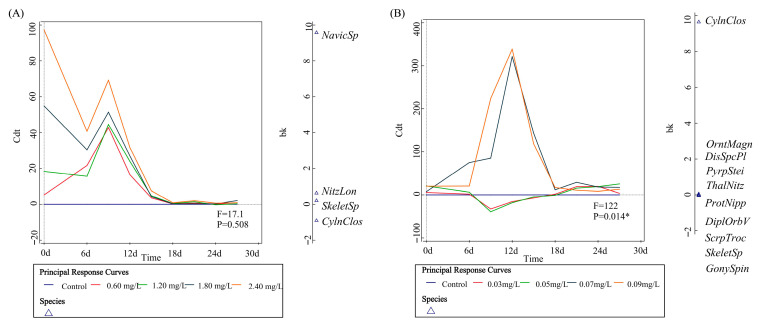
PRC results on the effects of DIN (**A**) and SRP (**B**) on the species composition of phytoplankton communities. Note: Cdt represents the degree to which the composition of phytoplankton species at each SRP concentration deviates from the control, and bk represents the degree to which species contribute to PRC. * *p*-value < 0.05.

Figure 2 and Figure 5 illustrate that in the DIN treatment group, the Shannon–Wiener Diversity Index, Pielou’s Evenness Index, and Margalef’s Richness Index of the phytoplankton community exhibited a considerable fall as DIN concentration increased, with the reduction becoming increasingly evident over time. This pattern suggests that the rise in DIN adversely affects the composition of the phytoplankton population, resulting in a notable decline in diversity, evenness, and richness. On the 30th day, the diversity indices of all treatment groups were markedly lower than those of the control group, with the most pronounced reduction observed in the high-concentration group. This indicates that the rise in DIN results in a more simplistic and less stable phytoplankton community structure. Multivariate analysis (PRC) of the phytoplankton community indicated that during the initial phase of microcosm development (prior to 15 days), DIN treatment substantially modified the species makeup of the phytoplankton. After 15 days, the species composition remained steady compared to the control group, indicating both the short-term reaction and long-term adaptation of the phytoplankton community to DIN enrichment. Initially, species susceptible to DIN may proliferate rapidly, resulting in alterations in species composition, but over time, competition within the community and interspecies interactions stabilize, rendering the community response smoother. *Navicula* sp. was the primary contributor to alterations in community composition, whereas the trends in *Skeletonema* and *Nitzschia serialis* exhibited an inverse relationship to the total community changes.

### 3.4. Effects of SRP on Phytoplankton Community Structure

Figure 4 and Figure 5 illustrate that the Shannon–Wiener Diversity Index, Pielou’s Evenness Index, and Margalef’s Richness Index of the SRP treatment group markedly declined over time, signifying a substantial adverse effect of escalating SRP on the phytoplankton community structure. At the conclusion of the experiment, the community diversity, evenness, and richness in the various SRP concentration treatment groups significantly diminished in comparison to the control group, indicating a substantial detrimental effect of elevated SRP on the phytoplankton community structure. Multivariate analysis (PRC) of the phytoplankton community indicated that soluble reactive phosphorus (SRP) significantly influenced the species makeup of the plankton community (*p*-value = 0.014). Between the 6th and 18th days of construction, elevated concentrations of SRP markedly influenced species composition, but lower concentrations of SRP exerted modest effects on species composition. *Nitzschia serialis* had the greatest impact on community composition alterations, but most algae species, including *Skeletonema* and the ovate variant of *Prorocentrum*, contributed minimally to these modifications.

### 3.5. Ecological Response Thresholds of DIN in Microcosm

**Figure 6:**
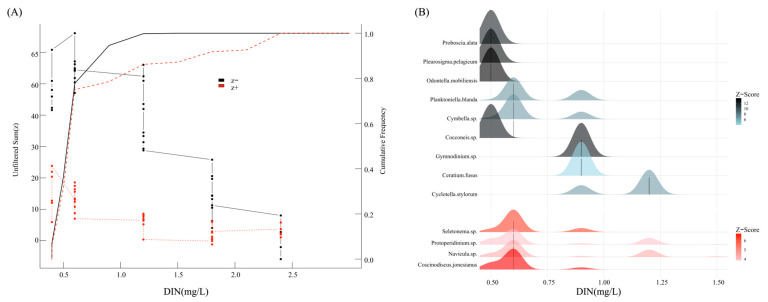
The response curve (**A**) and indicator species (**B**) of phytoplankton communities identified by TITAN analysis to DIN.

Phytoplankton, as direct consumers of nutrients, exhibit a community structure directly linked to the amounts of dissolved inorganic nitrogen (DIN) and soluble reactive phosphorus (SRP). TITAN analysis can ascertain the DIN and SRP thresholds that instigate alterations in phytoplankton community species. Figure 6 illustrates that the negative response threshold and positive response threshold of the phytoplankton community to DIN are 0.50 mg/L and 0.60 mg/L, respectively. When the DIN concentration is below 0.50 mg/L, the community species composition remains relatively stable; however, at 0.50 mg/L, sensitive species experience stress, and as the DIN concentration increases, species with greater tolerance to DIN gradually dominate. This study identified 9 negative response species and 4 positive response species as indicator species, based on the TITAN analysis. *Cocconeis* sp., *Odontella mobiliensis*, *Pleurosigma pelagicum*, and *Proboscia alata* were the most sensitive, negatively responding species to DIN, whilst the other algae exhibited more resistance to DIN. In the positively responding species, the prevalence of *Skeletonema* sp., *Protoperidinium* sp., *Navicula* sp., and *Coscinodiscus jonesianus* exhibited a significant upward trend with increasing DIN concentration, suggesting that they possess a heightened competitive advantage in environments characterized by elevated DIN levels. *Skeletonema* and *Prorocentrum* are primarily recognized as the principal species responsible for red tides, and they also dominate in the Yangtze Estuary [33].

### 3.6. Ecological Response Thresholds of SRP in Microcosm

**Figure 7:**
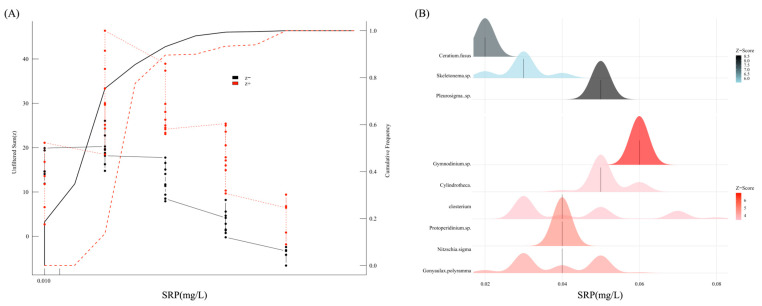
The response curve (**A**) and indicator species (**B**) of phytoplankton communities identified by TITAN analysis to SRP.

The negative response threshold and positive response threshold of phytoplankton communities to SRP are 0.03 and 0.04 mg/L, respectively. In addition, based on TITAN, three negative response species and five positive response species were identified (Figure 7). Among the negatively responsive species, *Ceratium fusus* was the most sensitive to SRP, while *Skeletonema* sp. and *Pleurosigma* sp. showed a greater tolerance to SRP. In the positively responding species, the abundance of *Protoperidinium* sp., *Nitzschia sigma*, *Cylindrotheca closterium*, and *Gymnodinium* sp. all increased with rising SRP concentrations, indicating that they possess a stronger competitive advantage at high SRP levels.

Note:(z−) is the negative response species, corresponding to blue in (B); (z+) is the positive response species, corresponding to red in (B). The higher the mountain, the more concentrated the data; the darker the color, the stronger the response.

Note (z−) is the negative response species, corresponding to blue in (B); (z+) is the positive response species, corresponding to red in (B). The higher the mountain, the more concentrated the data; the darker the color, the stronger the response.

## 4. Discussion

### 4.1. Phosphorus-Induced Shift from Diatoms to Dinoflagellates

This study examined the long-term (30-day) impacts of DIN and SRP on phytoplankton populations at the community level and mimicked the continuity of nitrogen and phosphorus nutrient fluxes in actual water bodies using a constant concentration design. In contrast to conventional cultivation studies that utilize a singular application of nitrogen and phosphorus, the constant concentration approach more precisely simulates the growth and reproduction of phytoplankton under continuous nutrient supply conditions. The constant concentration approach facilitates accelerated algae multiplication and division, thus more explicitly illustrating species-level alterations within the community. This method improves both the sensitivity and accuracy of the experiment while offering a more thorough understanding of the dynamic processes via which phytoplankton communities react to nutritional salts [34]. This strategy has enabled us to acquire more valuable information, encompassing alterations in the composition, variety, evenness, and richness of phytoplankton communities.

The influence of nutrients on the composition of phytoplankton communities in estuarine regions differs among groups [35,36]. Different species of phytoplankton, owing to their distinct nutrient requirements and metabolic pathways, demonstrate varied response mechanisms under identical nutritional conditions [37,38,39]. This study revealed that the development rate of diatoms markedly escalated with increasing concentrations of DIN, indicating a positive response to nitrogen. Conversely, the abundance of dinoflagellates exhibited a declining trend over time, particularly noticeable in environments with elevated DIN concentrations. In addition, this article used the TITAN method to screen out four algae species that responded positively to DIN input, all of which were diatoms. A comprehensive analysis of the influence of nitrogen and phosphorus nutrients on the composition of phytoplankton communities was performed in the Jiaozhou Bay region, showing that nitrogen enrichment markedly enhanced diatom cell proliferation [40]. This discovery aligns with the study’s conclusions, suggesting that nitrogen is more crucial for diatom growth in estuarine regions. This is partly because diatoms have a strong adaptability to environmental changes and can quickly respond to changes in DIN concentrations, adjusting their metabolic activities to support growth [41]. On the other hand, diatoms possess a more efficient nitrogen assimilation mechanism, and during their growth, they can even convert some DIN into organic nitrogen through assimilation, further promoting their own growth and reproduction [42]. These features allow diatoms to demonstrate significant competitive prowess when nitrogen sources are plentiful, leading to fast proliferation and dominance in the aquatic environment. In the SRP treatment group, the increase in nutrients effectively promoted the rapid proliferation of diatom biomass during the initial phase of the experiment, significantly increasing their proportion in the phytoplankton community and illustrating the effective utilization and competitive superiority of diatoms in nutrient acquisition. However, during the mid and late phases of the experimental culture, the community progressively transitioned towards dinoflagellates and cyanobacteria, accompanied by a significant decline in diatom abundance. The TITAN method-selected algal species that exhibited a positive response to SRP further substantiated this phenomenon. Of the five algal species that showed a positive response, only one was a diatom, with the remaining four being dinoflagellates. The response value for this diatom was relatively modest, at 0.04 mg/L. At higher concentrations of SRP, the abundance of dinoflagellates increases more significantly. This occurrence offers potential evidence for the transition of plankton from diatom dominance to dinoflagellate dominance in the Yangtze Estuary in recent years. Prolonged, high-concentration phosphorus input causes a transition in the estuarine plankton population from diatom dominance to dinoflagellate dominance [43]. This study also revealed that in both the DIN and SRP treatment groups, variations in diatom abundance were closely correlated with the overall trends in phytoplankton abundance. This could be because the original water used for the microcosm experiment in this study was collected from the Yangtze Estuary, where diatoms have always been the dominant algae, accounting for a large proportion of phytoplankton, and thus their abundance has a direct impact on the total abundance of phytoplankton [44,45,46]. In the microcosm systems, greater concentrations of DIN and SRP correlated with a decline in the diversity, evenness, and richness of the phytoplankton community, suggesting that elevated nutrient levels render the phytoplankton community less complex and more unstable [47]. This may result from excessive nutrient input, fostering circumstances for the over-proliferation of some algae, which occupy greater ecological space and deplete significant amounts of oxygen. The aforementioned event results in heightened turbidity in the body of water and induces hypoxia. The hypoxic conditions and rampant algal proliferation jeopardize the survival of other phytoplankton, thereby impacting the variety of the plankton community [6,48,49]. Furthermore, the structural alterations in the phytoplankton population noted in the experiment may significantly impact the functionality and stability of the ecosystem. The decline in community diversity may impair the ecosystem’s capacity to withstand external perturbations, whereas alterations in community evenness may influence material cycling and energy movement within the ecosystem [50].

In addition to the absolute concentrations of nitrogen and phosphorus nutrients, the ratio of nitrogen to phosphorus also affects the structure of plankton communities [51,52,53]. The Redfield ratio indicates that when the nitrogen-to-phosphorus ratio in a water body surpasses 16, phosphorus becomes the limiting nutrient; conversely, nitrogen is the limiting nutrient if the ratio is lower [54]. In this study, the nitrogen-to-phosphorus ratio in the DIN treatment group ranged from 60 to 240, indicating a pronounced phosphorus limitation feature. Under conditions of high nitrogen-to-phosphorus ratios, diatom growth was markedly enhanced with increasing DIN concentrations, but dinoflagellate abundance diminished with time, particularly at high DIN levels. The nitrogen-to-phosphorus ratio in the SRP treatment group varied from 4 to 13, indicating a nitrogen restriction, signifying that nitrogen is a more limited resource compared to phosphorus. In conditions of nitrogen limitation, the population of diatoms progressively diminished in the later phases of the experiment, while dinoflagellates and cyanobacteria continued to increase. Prior research has predominantly indicated that dinoflagellates exhibit a degree of insensitivity to temperature and nutrient salts, favoring habitats characterized by low phosphorus levels and high nitrogen-to-phosphorus ratios [42,55,56]. Nevertheless, certain studies have discovered that elevated nitrogen-to-phosphorus ratios facilitate diatom proliferation, whereas cyanobacterial blooms are more prone to manifest in hyper-eutrophic aquatic environments characterized by lower nitrogen-to-phosphorus ratios [57]. The findings of this study align more closely with the latter, suggesting that dinoflagellates can secure a competitive edge in interspecific interactions during the latter phases, even in environments with low nitrogen-to-phosphorus ratios. The observed variances are likely attributable to variations in the research location, the physicochemical circumstances of the experiment, and the disparities among the species examined.

### 4.2. Ecological Response Thresholds Lower than Stress-Response Values

This research utilizes the TITAN method in a microcosm to examine the quantitative link between phytoplankton and nitrogen and phosphorus nutrients. Table 3 presents the defined thresholds for the positive and negative responses of the phytoplankton community structure to dissolved inorganic nitrogen (DIN) and soluble reactive phosphorus (SRP). The negative response threshold signifies the pivotal concentration at which the population of sensitive species diminishes, instigating alterations in community structure, thereby profoundly affecting the phytoplankton community’s structure, function, and overall aquatic ecosystem [58,59]. Consequently, to preserve the stability of the phytoplankton community structure and to optimize the safeguarding of the majority of phytoplankton in estuarine water bodies against nutrient fluctuation disturbances, this study employs the negative response threshold as the ecological response threshold. Our research group previously derived response thresholds for DIN and SRP based on environmental monitoring data from 2010 to 2022 using the stress–response model method. The response thresholds for DIN and SRP derived using Chl a as the response variable are 1.643 and 0.065 mg/L, respectively, and those derived using DO as the response variable are 2.173 and 0.052 mg/L, respectively, all of which are higher than the response thresholds in this study [60]. The ecological response thresholds in this study focus on preserving community structure stability, establishing the response values of sensitive species as thresholds, resulting in lower values. Prior studies concentrated on the total Chl a concentration and dissolved oxygen levels, representing community abundance and oxygen consumption, without considering the stability of community structure. These secondary ecosystem responses may exhibit lag effects, resulting in higher response values. Furthermore, prior research relied on in situ monitoring of water chemistry data, with numerous factors influencing variations in nitrogen and phosphorus concentrations, including terrestrial inputs, atmospheric deposition, marine tides, freshwater runoff, water residence time, wind, and suspended particulate matter, among others [49,61]. Consequently, the monitoring data are affected by multiple causes, resulting in uncertainties in the estimated response thresholds.

### 4.3. Strengths, Limitations, and Recommendations

This study markedly contrasts with the findings of monoculture tests. Monoculture investigations frequently establish ideal growth conditions for algae, including optimized temperature, light, and salinity parameters, to enhance their growth rate and biomass [43]. This method fails to accurately represent the reality of multispecies interactions in intricate natural ecosystems. This study optimizes the restoration of the background ecological attributes of the study area. This method facilitates a thorough comprehension of the complexity and diversity of ecosystem responses while providing substantial guidance for the determination of ecological response thresholds. Despite these strengths, several limitations need to be acknowledged. The absence of grazers in our microcosm systems means that we did not account for the potential impacts of zooplankton predation on phytoplankton dynamics [62]. Grazers can significantly influence phytoplankton community structure and biomass, and their exclusion may have led to an overestimation of phytoplankton growth and response to nutrient enrichment [63]. The water volume in our experiment was relatively small, and constant temperature and lighting conditions were maintained during the experiment. These conditions cannot fully reflect the natural changes of these factors in the estuarine environment and may affect the scalability of our results in larger estuarine ecosystems. Moreover, although this work mitigated the effects of nutrient concentration drop on experimental outcomes by maintaining constant concentrations, the availability of nutrients in natural water bodies may still be affected by numerous factors, including water flow and sediment discharge [64,65]. Consequently, future research should aim to incorporate grazers into experimental designs and consider more dynamic environmental conditions to better understand the complex interactions between nutrients, phytoplankton, and other biotic and abiotic factors in estuaries. This will help to more precisely model and forecast alterations in phytoplankton communities in seawater.

## 5. Conclusions

This research established constant concentration aquatic microcosm systems and examined the prolonged (30-day) impacts of DIN and SRP on the phytoplankton at the community level. The results indicated that elevated nutrient concentrations could enhance the overall abundance of phytoplankton, leading to variations in abundance among phytoplankton taxa and resulting in increased levels of Chl a. The PRC demonstrated that elevated nutrient concentrations could modify community species composition to differing extents. Extended exposure to elevated levels of DIN fosters diatom proliferation and suppresses dinoflagellate development, whereas prolonged exposure to high concentrations of SRP encourages dinoflagellate growth and hinders diatom proliferation. Sustained high phosphorus inputs have caused a planktonic community shift from diatoms to dinoflagellates. These combined changes ultimately lead to an overall decline in community diversity indices, including the Shannon–Wiener Diversity Index, Pielou’s Evenness Index, and Margalef’s Richness Index, thereby simplifying and destabilizing the phytoplankton community structure. The TITAN method analysis revealed that the phytoplankton community exhibits a negative response threshold to DIN at 0.50 mg/L and a positive response threshold at 0.60 mg/L, identifying 9 species with negative responses and 4 species with positive responses as indicators. For SRP, the negative response threshold is 0.030 mg/L, and the positive response threshold is 0.040 mg/L, also identifying 3 negative response species and 5 positive response species as indicators. To safeguard the majority of phytoplankton species against nutrient perturbations, the negative response threshold is adopted as the ecological threshold—0.50 mg/L for DIN and 0.030 mg/L for SRP. These values are equal to the current Class IV and Class II limits of seawater quality standards, respectively. This indicates that for the Yangtze Estuary, the existing DIN restrictions are too lenient, approaching the critical threshold for significant response of phytoplankton and increased risk of eutrophication, while SRP restrictions are relatively strict, consistent with ecological thresholds. Therefore, to better prevent coastal eutrophication, the DIN standard should be tightened, while SRP control should be maintained or moderately strengthened.

This work elucidates the influence of nutrient concentrations on the composition and variety of phytoplankton communities, offering scientific insights for aquatic ecosystem management and ecological preservation. Future research should continue to examine the intricate relationships between nutrients and aquatic environments to enhance ecosystem health and sustainability.

## Figures and Tables

**Table 1:** DIN concentration settings.

Experimental Group	DIN (mg/L)	SRP (mg/L)
Control group 0.4 mg/L	0.4 mg/L	0.010 mg/L
DIN 0.6 mg/L	0.6 mg/L	
DIN 1.2 mg/L	1.2 mg/L	
DIN 1.8 mg/L	1.8 mg/L	
DIN 2.4 mg/L	2.4 mg/L	

**Table 2:** SRP concentration settings.

Experimental Group	SRP (mg/L)	DIN (mg/L)
Control group 0.010 mg/L	0.010 mg/L	0.40 mg/L
SRP 0.030 mg/L	0.030 mg/L	
SRP 0.050 mg/L	0.050 mg/L	
SRP 0.070 mg/L	0.070 mg/L	
SRP 0.090 mg/L	0.090 mg/L	

**Table 3:** Establishment of ecological response values in microcosmic experiments.

Nutrient	Negative Response Threshold (mg/L)	Positive Response Threshold (mg/L)	Ecological Response Threshold (mg/L)
DIN	0.50	0.60	0.50
SRP	0.030	0.040	0.030

## Data Availability

The raw data supporting the conclusions of this article will be made available by the authors on request.
